# Case Report: post-stroke rehabilitation with a visuomotor transformation-based brain-computer interface

**DOI:** 10.3389/fnhum.2026.1774409

**Published:** 2026-04-14

**Authors:** Alisa Kokorina, Nikolay Syrov, Lev Yakovlev, Mikhail Lebedev

**Affiliations:** 1 Vladimir Zelman Center for Neurobiology and Brain Rehabilitation, Skolkovo Institute of Science and Technology, Moscow, Russia; 2 Faculty of Mechanics and Mathematics, Lomonosov Moscow State University, Moscow, Russia; 3 I.M. Sechenov Institute of Evolutionary Physiology and Biochemistry, Russian Academy of Sciences, Saint Petersburg, Russia

**Keywords:** brain-computer interface, motor imagery (MI), P300 - event related potential, rehabilitation, virtual reality

## Abstract

Brain–computer interfaces (BCIs) are increasingly explored as tools for post-stroke neurorehabilitation. Motor imagery (MI)-based paradigms are widely used but may be difficult for some patients to perform reliably, motivating the exploration of alternative control strategies. This study presents a retrospective exploratory case series (*n* = 5) evaluating the feasibility and safety of a P300-based BCI paradigm designed to engage visuomotor transformation processes during upper limb rehabilitation. Two patients underwent rehabilitation using the P300-based paradigm, while three patients used an MI-based BCI within the same rehabilitation framework. In both conditions, BCI control was integrated with a robotic orthosis and an immersive virtual reality (VR) environment. BCI performance, neurophysiological responses (event-related potentials and event-related desynchronization), and clinical measures (Fugl–Meyer Assessment of the Upper Extremity, NIHSS) were assessed before and after a 10-session rehabilitation course. All participants were able to achieve BCI control above chance level. Across cases, changes in clinical scores and consistent neurophysiological patterns associated with task engagement were observed. No adverse events or clinically significant safety concerns were identified. These findings suggest that a P300-based BCI paradigm incorporating visuomotor transformation can be feasibly implemented within a VR-assisted robotic rehabilitation framework. Given the exploratory design, small sample size, and heterogeneity of the cohort, the results should be interpreted as hypothesis-generating. Further controlled studies are required to determine the clinical relevance and potential applications of this approach.

## 1 Introduction

Brain–computer interfaces (BCIs) are increasingly explored in medical applications ranging from neurorehabilitation to communication and cognitive assessment (Liu et al., 2025). Brain-computer interfaces (BCIs) have emerged as a promising tool in the field of neurorehabilitation, including post-stroke rehabilitation (Mane et al., 2020).

ERP-based control paradigms, particularly those relying on the P300 component, continue to demonstrate feasibility for motor-independent interaction, although substantial inter-individual variability remains a key challenge (Hasegawa and Watanabe, 2025).

By engaging the brain regions involved in motor control, BCIs have the potential to induce neural plasticity and restore the functioning of brain circuitry affected by stroke. As such, BCIs hold promise of improving motor recovery and speeding up the overall rehabilitation process (Cervera et al., 2018).

Currently, motor imagery (MI) remains one of the most widely utilized BCI-based approaches in neurorehabilitation (Ang et al., 2010; Khan et al., 2020; Liu et al., 2025). MI evokes changes in brain activity as users mentally focus on kinesthetic sensations associated with imagined voluntary movements (Pfurtscheller and Neuper, 1997; Neuper et al., 2006). Despite the potential benefits of MI BCIs, achieving satisfactory accuracy and vividness requires proper training to control cortical rhythms by generating MI, which is difficult for many post-stroke patients (Sirigu et al., 1996; Malouin et al., 2007; Hougaard et al., 2021).

**Figure 1:**
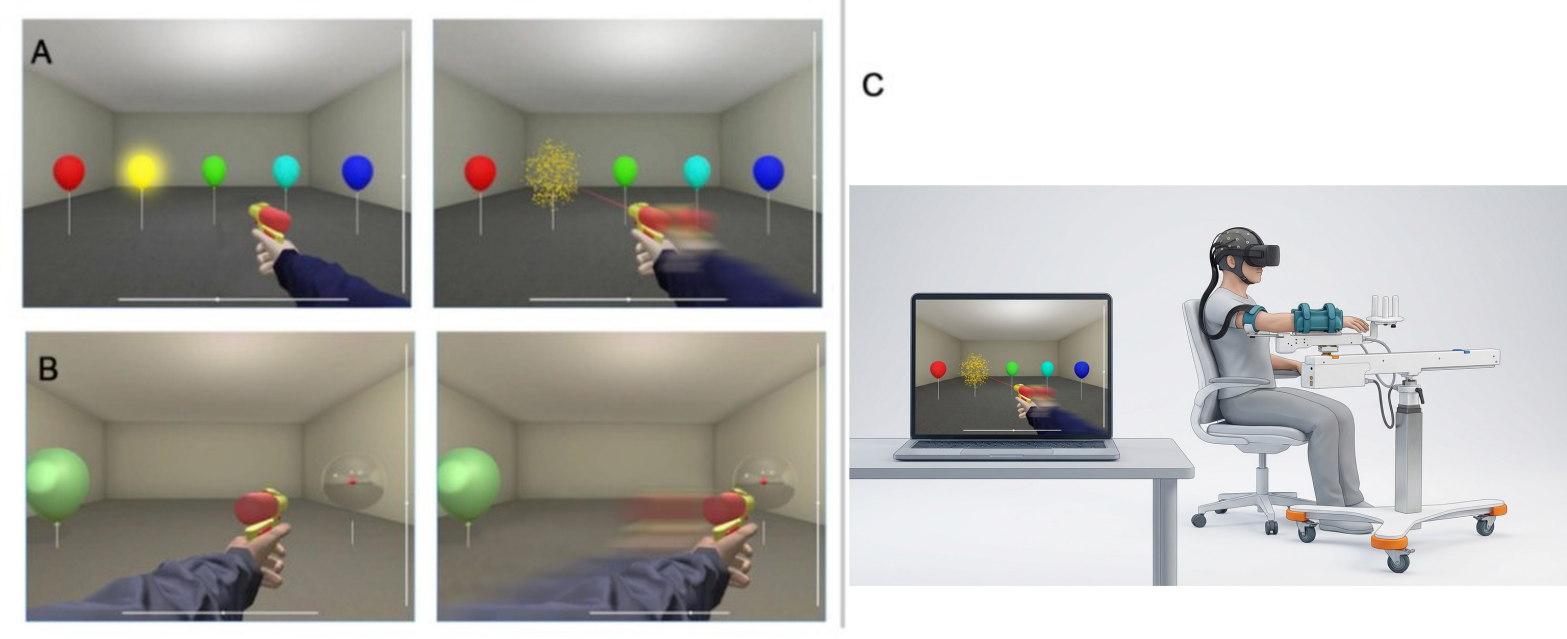
Experimental paradigms and setup. **(A)** Graphical interface of the P300-based paradigm in VR: target flash and subsequent movement of the limb toward the target balloon. **(B)** Graphical interface of the MI-based paradigm in VR: the limb moves toward the target balloon when MI-related changes in EEG are detected. **(C)** Experimental setup showing the participant seated with the paretic limb mounted on an assistive robot and wearing a VR headset.

In the BCI approach to post-stroke rehabilitation described here, we focused on the restoration of visuomotor transformations instead of relying on MI. Visuomotor transformation is the neural process where a visual stimulus (e.g., vision of a cup of coffee) undergoes transitions in multiple brain areas and eventually triggers a movement (e.g., picking the cup). This transformation engages both cortical and subcortical areas, each representing information in different coordinate frames (retinotopic, allocentric, muscle-based, etc.). Stroke can damage different parts of the visuomotor transformation circuitry, so an individual-based approach should be applied when designing a BCI that connects to this circuitry in an attempt to extract useful neural brain signals. Here, we employed a P300 paradigm to develop a BCI rehabilitation approach that enables a visuomotor transformation. In this approach, visual objects that serve as target stimuli for a P300 BCI are targets of movements that trigger visuomotor transformations. In addition to enabling a visuomotor transformation, this BCI design was intended to provide an alternative control strategy for patients who experience difficulty with MI-based BCIs. Indeed, even when multiple potential targets (Figure 1A) are present in the visual scene, P300-based BCIs have demonstrated robust classification performance across multi-target paradigms, such as speller systems (Fazel-Rezai et al., 2012). Furthermore, the BCI was integrated with a rehabilitation robot and virtual reality (VR) (Figure 1C), to provide multimodal feedback relevant for motor recovery. With this design, both somatosensory and visual feedback are present for limb movement (Syrov et al., 2023). The usage of robotic orthoses and VR systems has been developing for rehabilitation purposes. Integration of these systems with BCIs could be especially effective because of the embodiment of the virtual body parts (Duan et al., 2021).

In this study, several post-stroke patients underwent rehabilitation aided with a P300-BCI that combines a robotic orthosis with a VR environment. The participants directed their attention to flashes of the target object (Figure 1A) while ignoring the flashes of non-targets in virtual reality (VR) settings. They responded mentally to these flashes, anticipating the eventual robot-assisted reaching toward that object. In the MI paradigm (Figure 1B), participants concentrated on the target object, envisioning limb movement toward it. Upon successful classification, the BCI system triggered a robotic orthosis to move the affected limb toward the target. This movement mainly involved arm movement in the horizontal plane. To sum up, this approach offered visual and kinesthetic feedback of the robotically assisted movement, while the P300 BCI engaged attention and triggered a visuomotor transformation. As such, this BCI mimicked the essential stages involved in visually-guided arm movements and served as a rehabilitative system that provided an alternative control modality that does not rely on motor imagery, which can be challenging for some patients.

## 2 Materials and methods

### 2.1 Participants

This study represents a retrospective exploratory case series based on previously collected clinical data. The reporting of this work follows CARE guidelines where applicable. However, due to the heterogeneity of clinical documentation, some checklist elements (including a unified timeline, complete physical examination details, and formal patient-reported perspectives) were not consistently available across all cases. All patient data were de-identified prior to analysis. Several recruited patients were excluded due to poor EEG signal quality, excessive artifacts, or incomplete protocol execution.

Five paralyzed patients (all male, aged 40–66 years old) affected by cerebral stroke participated in this study (Table 1). The study protocol was approved by the Institutional Review Board of the Skolkovo Institute of Science and Technology (protocol No 4, 30.09.2021) and the review boards of the Moscow Department of Health and Federal Center of the Brain and Neurotechnologies.

**Table 1:** Patient-level characteristics, BCI performance, and clinical outcomes (n = 5).

Variable	Case 23	Case 26	Case 31	Case 32	Case 42
Demographics & Clinical					
Age	55	66	59	40	58
Sex	M	M	M	M	M
Diagnosis/Lesion Location	Cerebral stroke of the central branch of the right middle cerebral artery	Cerebral stroke of the left middle cerebral artery	Cerebral stroke of the right middle cerebral artery	Cerebral stroke in the vertebro-basilar system	Cerebral stroke of the right middle cerebral artery
Time Since Stroke (months)	3	3	5	2	1
Intervention					
BCI Paradigm	P300	P300	MI	MI	MI
Rehabilitated Hand	Left	Right	Left	Left	Left
Sessions Completed	10	10	10	10	10
BCI Performance					
Classification Accuracy	0.63	0.60	0.73	1.00	1.00
Cohen's Kappa	0.66	0.63	0.52	0.70	0.85
Sensorimotor Activation Localization (μ/β)	Parietal (Pz–P3)/central left hemisphere (C3–P3)	Central left hemisphere (C3)/central left hemisphere (Cz–C3)	Central left hemisphere (C3–P3)/central left hemisphere (C3–P3)	Central right hemisphere (C4)/central right hemisphere (C4)	Bilateral/central right hemisphere (C3)
Clinical Outcomes					
NIHSS (baseline)	5	6	11	9	10
NIHSS (post-intervention)	5	6	11	8	8
Δ NIHSS	0	0	0	−1	−2
FMA-UE (baseline)	20	28	0	18	14
FMA-UE (post-intervention)	40	40	11	26	26
Δ FMA-UE	+20	+12	+11	+8	+12
MoCA (baseline)	22	26	26	26	28
MoCA (post-intervention)	22	26	26	26	28
Δ MoCA	0	0	0	0	0
HADS-A (baseline)	1	8	8	4	1
HADS-A (post-intervention)	1	6	4	4	1
Δ HADS-A	0	−2	−4	0	0
HADS-D (baseline)	5	8	7	6	3
HADS-D (post-intervention)	3	6	4	4	2
Δ HADS-D	−2	−2	−3	−2	−1
mRS (baseline)	3	3	4	4	3
mRS (post-intervention)	3	2	4	3	3
Δ mRS	0	−1	0	−1	0
MRC (mean across upper limb muscle groups)					
MRC (baseline)	1.1	2.6	0.0	1.6	1.8
MRC (post-intervention)	2.2	2.6	1.0	2.0	2.6
Δ MRC	+1.1	0.0	+1.0	+0.4	+0.8

This table summarizes demographic and clinical characteristics, intervention parameters, neurophysiological BCI performance metrics, and clinical outcomes for all included patients. All participants underwent a standardized BCI-based rehabilitation protocol consisting of 10 sessions over approximately two weeks.

Clinical outcomes are presented as baseline and post-intervention values, along with absolute changes (Δ), and include neurological status (NIHSS), upper limb motor function (FMA-UE), cognitive performance (MoCA), emotional state (HADS-A and HADS-D), and functional disability (mRS). Muscle strength is reported as the mean Medical Research Council (MRC) scores across assessed upper limb muscle groups.

BCI performance is characterized by classification accuracy, Cohen's kappa, and sensorimotor activation localization in the μ and β frequency bands.

### 2.2 Scales

Several neurological scales were used to assess post-stroke rehabilitation, each of which provides a different measure of the patient's functional abilities and recovery:

- The National Institutes of Health Stroke Scale (NIHSS; Brott et al., 1989) measures the severity of stroke and the resulting neurological deficits. It includes assessments of consciousness, vision, motor function, sensation, language, and neglect.
- The Fugl-Meyer et al. (1975) assessment of the Upper Extremity (FMA-UE) measures motor function, balance, sensation, and joint function. It assesses the patient's ability to perform a range of movements, such as reaching, grasping, and releasing objects.
- Montreal Cognitive Assessment (MoCA; Nasreddine et al., 2005) assesses the patient's cognitive function, including attention, memory, language, and visuospatial abilities.
- HADS (Hospital Anxiety and Depression Scale; Zigmond and Snaith, 1983) is the anxiety and Depression assessment: HADS is a self-assessment scale designed to measure anxiety and depression levels in patients. Post-stroke patients commonly experience psychological distress due to the physical and emotional impact of stroke.
- mRS (Modified Rankin Scale; van Swieten et al., 1988) is the Functional Outcome assessment. This scale is essential for evaluating the effectiveness of interventions and treatments, tracking improvements, and determining the impact of stroke on a patient's quality of life.
- MRC (Medical Research Council Scale; Medical Research Council, 1981) is a Muscle Strength assessment (MRC Shoulder Flexors/Extensors, Elbow Flexors/Extensors, Pronators/Supinators, Wrist Flexors/Extensors, Finger Flexors/Extensors). These individual assessments of muscle strength provide detailed information about the specific impairments a patient is facing. This granularity aids in designing targeted rehabilitation programs that focus on improving particular muscle groups, enabling a more comprehensive recovery process.

In summary, these scales were essential for a holistic assessment of post-stroke patients' physical and psychological wellbeing. They provide quantifiable measures of disability, muscle strength, and psychological distress, allowing healthcare professionals to tailor interventions, track progress, and make informed decisions about treatment strategies.

### 2.3 BCI system

**Figure 2:**
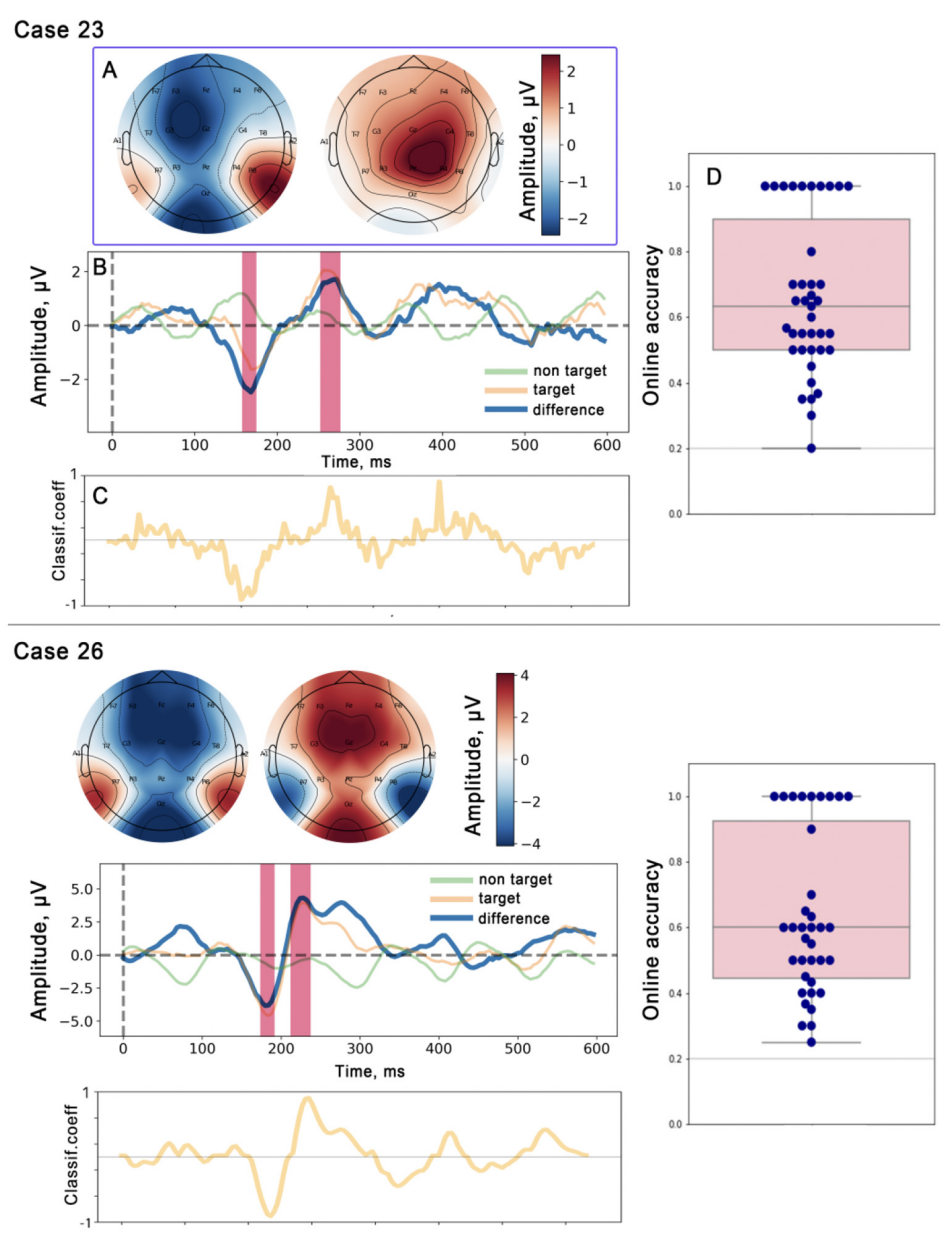
Results of EEG analysis during rehabilitation sessions with a robotic system controlled by a P300-based BCI. Data from two patients are presented. **(A)** Topographic distribution of N200 and P300 peaks in the difference curve: Left: N200 topographic distribution, Right: P300 topographic distribution. **(B)** Target and non-target ERPs from the Cz channel: averaging of 5 850 target epochs and 23 400 non-target epochs for Case 23 and 5 400 and 21 600 for Case 26. **(C)** Visualization of the classifier coefficients: The amplitude of the deviation from zero indicates the importance of the features in the classification task (target vs. non-target). **(D)** Distribution of online classification accuracy for the P300 paradigm. The box plots summarize the distribution of accuracy scores from all individual BCI runs for Case 23 (*n* = 39 runs) and Case 26 (*n* = 36 runs). Each dot represents the accuracy achieved in a single BCI run. The horizontal gray line indicates the theoretical chance level (0.2).

Two BCI paradigms were adopted for the rehabilitation system:

– The P300 paradigm, where the patient selected the target of movement using a P300 BCI, followed by a robotically assisted reaching movement of the upper limb, accompanied by VR feedback;
– The MI paradigm, where patients modulated their cortical μ- rhythms by imagining moving their upper limbs;

The patient was given the option to complete exercises throughout rehabilitation, with the program being displayed using virtual reality (VR) glasses. During exercises, the patient is required to use the paralyzed hand to imitate common movements, such as flexing and extending the arm at the elbow (Figure 2C). The brain-computer interface recognizes this movement by examining the patient's EEG.

### 2.4 Rehabilitation procedures

#### 2.4.1 The P300 paradigm

The P300-based rehabilitation started with an attention training session where the patient was asked to focus visual attention on the target object (an image of a balloon in VR) while it was flashing. This mode was necessary to assess the patient's cortical response and adjust the classifier for the upcoming rehabilitation session. Next, training was conducted where the target object was marked by an indicator that was filled with green color after the required concentration level was reached.

After the completion of this stage, the rehabilitation mode began, during which the target ball was slowly illuminated three times. After its detection, the robot assistant moved the patient's hand. The virtual hand with a laser gun moved to the selected object and “fired” at it (Figure 2A). One “shot” on the ball took about 15 s. 10 repetitions of the exercise were performed within one session. Each patient completed a rehabilitation course consisting of 10 clinical visits. Within each visit, multiple BCI-controlled exercises, hereafter referred to as “BCI runs”, were performed. The total number of BCI runs varied per patient and is reported in the results.

#### 2.4.2 The MI paradigm

During the MI-based rehabilitation, patients had to perform the MI of their paralyzed hand (depending on the individual case). Prior to the BCI-online sessions, there were 10 trials used for calibration of the system, in which MI trials were followed by resting state periods.

The simulator was first trained to understand the patient's desire to move the hand; at this point, the patient's hand is inside a robotic device, and the robot assistance moved the hand to one of the virtual reality balls by moving it to the left or right. The patient was focused on the warmth and power of the muscles in the hand as it moved, and during the pauses between movements, he or she relaxed and stopped picturing any movement at all. Ten movements to the left and ten to the right were made during the training session, which took around 5 min.

The MI-rehabilitation exercise involved two balloon targets placed on opposite sides of the visual area (one on the left and one on the right, Figure 2B). Patients were instructed to imagine their paralyzed hand moving toward one of the balloons (referred to as the target). Based on the quality of their motor imagery efforts, a bar next to the target balloon was filled. When the bar was completely filled, it resulted in the correct movement of the hand toward the target, and a “shot” at the target balloon was achieved. One attempt took about 30–40 s, and each exercise was completed several repetitions, while the entire session of rehabilitation lasted about 25–30 min.

### 2.5 Data acquisition

During the rehabilitation trials, a 20-channel monopolar EEG was recorded at 250 Hz using an NVX-36 DC amplifier (MKS, Zelenograd, Russia). Passive Ag/AgCl electrodes were positioned across the scalp at the following locations according to the international 10–20 system: *Fp1, Fp2, F7, F3, Fz, F4, F8, T7, C3, Cz, C4, T8, P7, P3, Pz, P4, P8, Oz,a1,a2*. For the MI sessions, 17 channels were used (*Fp1, Fp2*, and *Oz* were removed as non-target for the motor imagery). The reference electrodes were placed at *the right and left* mastoids, and the ground electrode was placed at the *aFz* position. The skin-electrode impedance for each electrode was below 20 kΩ. The recorded signal was filtered between 0.1 and 75 Hz using a FIR filter, with an additional 50 Hz Notch-filter. Raw data acquisition and stimulus presentation were carried out using the “VIBRaINT RehUp” software (Vibraint, Samara, Russia).

### 2.6 Data analysis

#### 2.6.1 Event-related potentials analysis

During the offline analysis of the evoked potentials acquired during rehabilitation sessions using the P300 paradigm, we performed EOG and blink artifact removal using the fastICA algorithm. Subsequently, the signal was band-pass filtered between 1 and 15 Hz using a fourth-order Butterworth filter, and a notch filter was applied to reduce line noise interference. The processed data were segmented into [−0.1 to 1] s epochs, aligned with the onset of the stimulus flashes, with the [−0.1 to 0] s interval for baseline correction. Each participant contributed a total of 3,750 target epochs and 15,000 non-target epochs.

To identify the ERP components most influential for classification, we used data from each rehabilitation session. We used stochastic gradient descent learning-based regularized linear model fitting (via the “SGDClassifier” function from the scikit-learn Python library, version 1.0.2). The signal from the Fz, Cz, and Pz channels was used. We chose L1 regularization with α = 0.5 to sparsify the feature vector by setting insignificant feature coefficients to zero, thereby contrasting features (epoch samples) that are critical for discriminating between targets and non-targets within the BCI control. Coefficients were then averaged over all BCI runs and channels used, followed by visualization of the resulting curve representing the value of the classifier coefficients for each time stamp of the epochs used in training.

To estimate online accuracy, we calculated the ratio of correct BCI outputs to the total number of trials performed within each session. To account for the chance level, which was 0.2 for 5 stimuli in the P300 paradigm and 0.5 for the MI-BCI, we used Cohen's Kappa accuracy assessment beyond simple accuracy.

#### 2.6.2 Event-related desynchronization estimation

For the event-related desynchronization (ERD) assessment, the raw data were bandpass (1–30 Hz; FIR filter) and spatial filtered via Common average Reference (following McFarland et al., 1997). Then, the signals were epoched (5-s epochs for both MI and Rest conditions). The frequency domain analysis was conducted using the Fast Fourier Transform (FFT) with a 1-s time-window and an 85% overlap, allowing for Power Spectral Density (PSD) estimation using the Welch method. To calculate the ERD, the PSD values were transformed into dB using the following formula: ERD = 10 ^*^ log10(PSD*mi/*PSD*rst*), where PSDmi represents the median power spectral density across the MI epochs, and PSDrst denotes the median power spectral density calculated for the resting state epochs. The ERD assessment was performed in individualized μ- and β- frequency ranges for each subject. The obtained ERD values for each channel were then used to plot topographic maps.

In the conditions with the P300-BCI control, we performed the time–frequency analysis of oscillatory activity changes during the robot-assisted paralyzed limb movement launched as BCI-feedback. We used a set of complex Morlet wavelets with a variable number of cycles for different frequencies. The frequencies of the wavelets ranged from 5 to 25 Hz with a 1 Hz step; the full-width at half-maximum (FWHM) was equal to 248 ms.

## 4 Results

Potential risks associated with the intervention included transient visual fatigue related to VR exposure and mild discomfort during system use; however, no serious adverse events or clinically significant complications were observed in this cohort.

### 4.1 Event-related potentials in P300 BCI control

In both patients, we observed a comparable temporal pattern of ERPs during P300 BCI control (Figure 2). The difference curve (representing targets minus non-targets) for the *Cz* channel showed a negative deflection peaking at approximately 200 ms latency (N200), followed by a subsequent positive wave in the 250–350 ms latency range (P300). In particular, the linear classifier with L1 regularization, trained on epochs from the *Cz* channel, showed higher coefficients within these specific temporal windows, indicating the contribution of the N200 and P300 components to BCI control. However, the topography differed between the two patients. In Case 26, both peaks show a fronto-central localization, whereas in Case 23, the N200 peak was localized in frontal areas with a slight lateralization to the left side, and the P300 peak showed a parietal predominance.

Both participants achieved online accuracy significantly above chance. For Case 23, the median accuracy across 39 BCI runs was 0.63, with perfect accuracy (1.0) achieved in 10 of these runs. For Case 26, the median accuracy across 36 BCI runs was 0.60, with perfect accuracy achieved in 9 runs (Figure 2D).

#### 4.1.1 Event-related desynchronization during paralyzed limb movement

**Figure 3:**
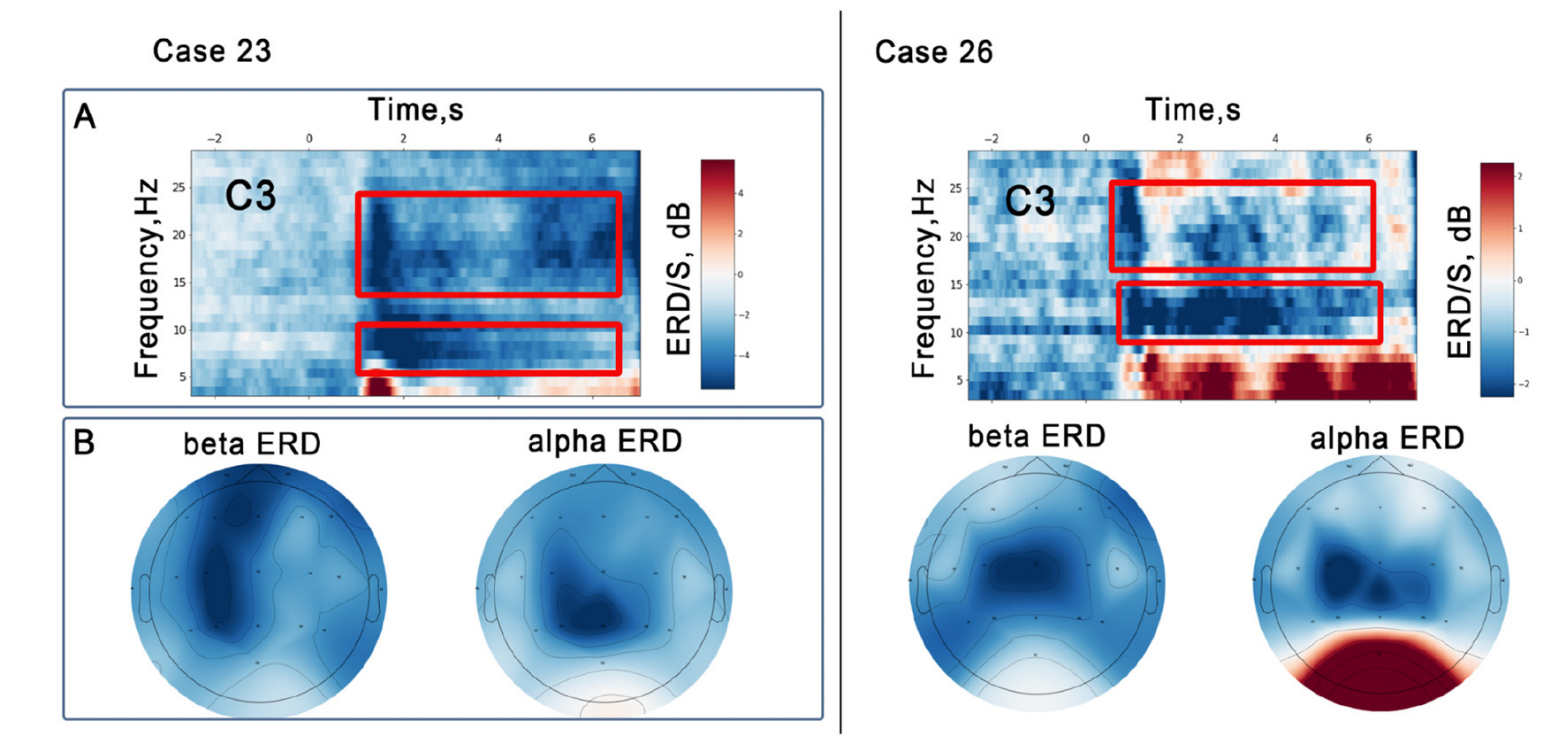
Results of EEG analysis during robotic movement of a paralyzed limb initiated as feedback in a P300 BCI session. Data from two patients are shown (Case 23, Case 26). **(A)** Averaged time-frequency dynamics of event-related ERD/S. Red rectangles indicate the time-frequency ranges used to estimate the topographic distribution. **(B)** Averaged topographic plots of μ- and β-rhythms during paralyzed limb movement. The color gradient indicates ERD/S values. Ipsilateral ERD patterns should be interpreted cautiously.

Following successful classification, the robotic system initiated movement of the paretic limb toward the target object. This passive movement induced a significant decrease in oscillatory activity in the α and β frequency ranges in both patients (Figure 3). The reduction in oscillatory activity was mainly observed over sensorimotor areas, with a particular predominance over the left hemisphere, which is ipsilesional in Case 26 but contralesional in Case 23. Also, in Case 23, the μ-ERD was more strongly focused in the parietal area (*Pz* channel) with only a slight tendency to the left side.

The temporal dynamics in the β-ERD showed rapid responses at the onset of robot movement, whereas the μ-ERD showed a longer temporal spread. Despite these temporal differences, both the μ- and β- ERD lasted for approximately 6 s from the onset of robot motion.

### 4.2 Event-related desynchronization in motor imagery BCI control

**Figure 4:**
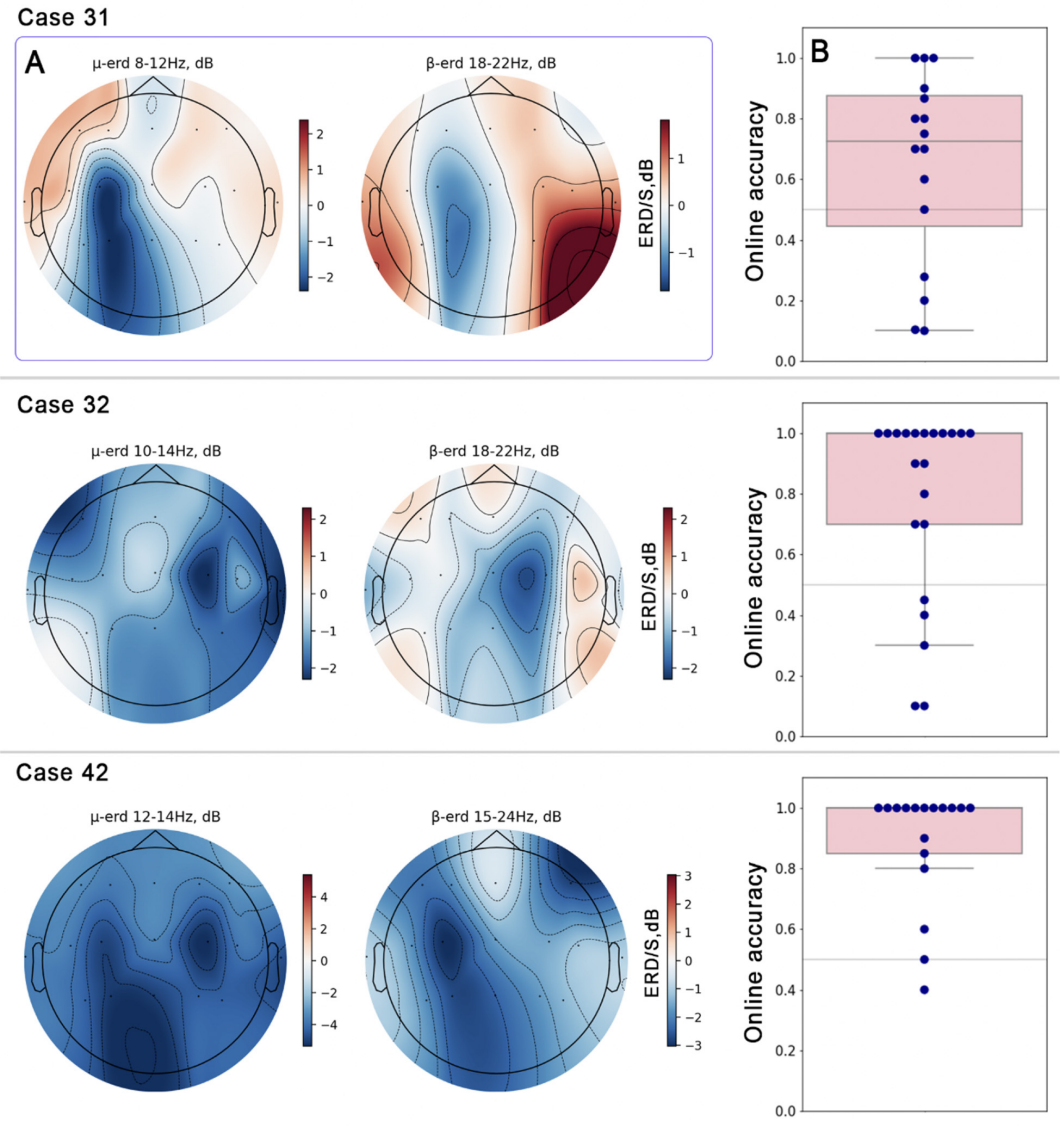
Results of EEG analysis during rehabilitation sessions with a robotic system controlled by MI-based BCI. Data from three patients are presented (Case 31, Case 32, Case 42). **(A)** Averaged topographical representations of the μ- and β-rhythm during motor imagery. The color gradient indicates ERD/S values. **(B)** Distribution of online classification accuracy for the MI paradigm. The box plots summarize the distribution of accuracy scores from all BCI runs for Case 31, Case 32, and Case 42. Each dot represents the accuracy achieved in a single BCI run. The horizontal gray line indicates the theoretical chance level (0.5).

During the MI-based rehabilitation, all three patients demonstrated proficiency in controlling the BCI via motor imagery (Figure 4). This stable ERD was accompanied by a good online BCI-classification accuracy across the rehabilitation sessions. The ERD was consistently located in the target C3/C4 channels (depending on the individual case). For Case 31 most prominent ERD was located in the left hemisphere (C3–P3 channels), both in μ- (10–14 Hz) and β- (18–22 Hz) ranges. The median classification accuracy was 0.73, which is higher than the chance level (0.5). For Case 32, there was a right-hemisphere located ERD, pronounced in the C4-channel in μ- (10–14 Hz) and β- range (18–22 Hz). Median classification accuracy for this case was 1.00. For Case 42, there was a bilateral ERD pattern localized in C3- and C4- channels for the μ -rhythm (12–14 Hz) and ERD in the β range (15–24 Hz) localized in the C4-channel. Classification accuracy obtained during rehabilitation BCI-sessions was 1.00.

### 4.3 Rehabilitation efficiency assessment

**Figure 5:**
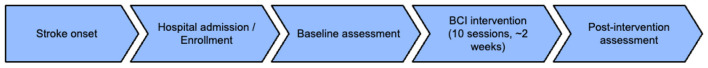
Study timeline. Schematic representation of the study design showing the sequence of clinical assessment and BCI-based rehabilitation. Patients were enrolled after stroke onset and underwent a baseline evaluation, followed by a standardized BCI-based intervention comprising 10 sessions over ~2 weeks, with a post-intervention assessment performed immediately after completion of training.

Clinical outcomes for the five participants were assessed before and after the BCI rehabilitation course, which consisted of 10 sessions per patient; a unified study timeline is provided (Figure 5). Detailed patient-level results are summarized in Table 1.

Changes in upper limb motor function were observed across all cases, as reflected by increases in FMA-UE scores ranging from +8 to +20 points. The largest gains were observed in Case 23 (+20) and Case 31 (+11), the latter starting from complete motor impairment (baseline FMA-UE = 0).

Neurological status (NIHSS) remained stable in three patients and changed in two cases (Cases 32 and 42), indicating no deterioration during the intervention period. Disability level (mRS) showed a change in one case (Case 26) and remained stable in others.

Muscle strength assessments (MRC scale across multiple upper limb muscle groups, detailed muscle-group-specific MRC scores are provided in Supplementary Table 1) demonstrated consistent increases across patients, particularly in distal muscle groups (wrist and finger flexors/extensors), suggesting distributed recovery of motor function rather than isolated gains.

Cognitive performance (MoCA) remained unchanged in all participants. Emotional state, assessed using HADS, showed partial improvement: anxiety scores decreased in two patients, while depression scores decreased in four out of five cases.

Overall, the results indicate consistent within-patient changes in motor and functional outcomes following the intervention, without evidence of cognitive decline.

## 5 Discussion

In this study, our goal was to introduce a rehabilitative framework including a VR environment with visually interactive objects and a robotic orthosis controlled by the patient's cognitive effort within a BCI. In addition to the motor-imagery paradigm commonly used in rehabilitation, our approach integrated the P300 BCI, the control of which required a cognitive response in the form of an intention to execute a movement upon the appearance of a target object.

This innovative paradigm offers opportunities for individuals with disabilities who have difficulty with vivid motor imagery. During P300-BCI sessions, we observed that patients achieved a level of accuracy that resulted in reliable feedback, i.e., the initiation of passive movement of the paretic limb. As a result, the P300-BCI may have bridged the gap between the mental intention to reach a visual target and the subsequent movement-related sensorimotor activation, providing the completion of the visuomotor transformation process. Below, we discuss the cortical activity dynamics we observed for both BCI paradigms in detail, focusing on the EEG markers of sensorimotor activation, and discuss the results of the motor and cognitive changes we observed in the patients after the rehabilitation course.

### 5.1 P300 paradigm results

Within the P300 BCI sessions, both patients demonstrated robust above-chance accuracy across all rehabilitation sessions, with some sessions having no errors in 10 trials. These results emphasize the potential of post-stroke patients with motor dysfunction to effectively control P300-based BCIs. Analysis of the coefficients of the fitted classifier revealed that meaningful ERP components, namely N200 and P300, played a crucial role in the classification task. These ERP components are similar to the well-known peaks demonstrated in studies of P300-based BCIs (McFarland et al., 2011; Basyul and Kaplan, 2015). Notably, analogous ERP shapes have also been described in studies with healthy participants in similar P300-based BCI paradigms with visuomotor tasks (Syrov et al., 2022).

A notable result emerged from our analysis of the changes in the dynamics of sensorimotor rhythms related to paretic limb movements provided by a robot as feedback within the P300 BCI control. For both patients, we observed a significant decrease in both μ- and β-frequency oscillations. Interestingly, in patient 23, who suffered a right hemisphere stroke, β-ERD showed a left lateralization, i.e., over the intact hemisphere (regardless of the parietal localization of μ-ERD, it also showed a tendency to be lateralized to the left). Thus, as increased ERD in sensorimotor rhythms is associated with activation in somatosensory and motor cortices, these findings suggest that movement of the paretic limb (patient 23) was associated with an increase in the ipsilateral hemisphere. This finding suggests a potential compensatory mechanism or neural reorganization within the intact hemisphere to support the motor control and sensorimotor processes associated with paretic limb movement within the BCI feedback. Similar results were obtained in Park et al. (2016), where the authors observed ipsilateral motor cortex activation in patients with stroke-affected sensorimotor areas during passive and imagery movements. The damaged sensorimotor areas were no longer capable of normal motor function, and therefore the unaffected contralesional motor cortex took over the function of the damaged area (Park et al., 2016; Démas et al., 2020). The parietal localization of μ-ERD may also be explained by plastic reorganization resulting in a shift in the functions of sensorimotor cortices in the parietal area. This suggestion is supported by a study (Basu et al., 2010), which reported motor cortical function in the left occipital cortex of a subject who suffered a left middle cerebral artery stroke. The authors observed right-hand muscle building induced by transcranial magnetic stimulation of the left occipital cortex and suppression of the occipitally distributed α rhythm during right-hand movements.

Conversely, in Case 26, we observed that the topography of the μ-ERD was localized over the sensorimotor areas of the affected hemisphere. This localization strongly suggests activation of surviving neural circuits within the affected hemisphere. This observation is consistent with the comparatively weaker magnitude of the ERD in this subject compared to Case 23, in which we hypothesized that the healthy ipsilateral area became involved in the control of the paretic limb. In Case 26, however, it is conceivable that certain sensorimotor networks remained functional on the paretic side. This interpretation is also consistent with the higher initial Fugl-Meyer scores observed in this patient. Thus, these findings illustrate that neural activation during passive movement of the affected limb depends on the specifics of the stroke-induced damage.

Thus, our study revealed a significant achievement: motor-impaired patients were able to effectively control the P300-BCI and elicit robotic-assisted movement of their paretic limb solely through mental effort. The observed activation of sensorimotor areas during passive movement and the observation of catching targets in VR support our hypothesis that the potential of the P300-BCI involving visuomotor transformation may facilitate task-related cortical engagement that could be relevant for rehabilitation processes.

### 5.2 Motor imagery-related activation

Motor imagery practice, *per se*, is a way to induce neural plasticity and improve motor performance (for review, see Ruffino et al., 2017; Ladda et al., 2021). A BCI system allows the user to estimate their mental efforts via neurofeedback, making MI-based BCI a promising tool for neurorehabilitation, as confirmed in numerous studies (Frolov et al., 2017; Tang et al., 2018; Wang et al., 2019).

During the MI-based rehabilitation, all three patients showed changes in the rehabilitation process, becoming proficient in their motor imagery practice using a BCI-controlled robotic system. Pronounced central ERD and classification accuracy higher than the chance level (Figure 4) were observed. According to the generally accepted view, sensorimotor ERD reflects the activation of the corresponding cortical areas (e.g., the primary motor cortex, M1, and the primary somatosensory cortex, S1; Pfurtscheller and Neuper, 1997). ERD manifests in different tasks such as motor preparation, execution, imagery (Pfurtscheller and Neuper, 1997; Neuper et al., 2006), tactile stimulation, and tactile imagery (Yakovlev et al., 2023). The level (or magnitude) of the sensorimotor ERD is associated with the complexity of the performing task and reflects the size of the neuronal ensemble involved in cortical processing (Boiten et al., 1992; Pineda, 2005).

Normally, in sensorimotor-involved tasks, ERD manifests itself in the contralateral hemisphere due to the anatomy of corticospinal pathways (Pfurtscheller and Neuper, 1997; Yakovlev et al., 2023). However, for certain movements, such as finger movements, bilateral ERD has been reported in the literature (Horenstein et al., 2009; Yuan et al., 2010; Paek et al., 2014; Vasilyev et al., 2016, 2017; Hasegawa et al., 2017). This phenomenon could be associated with the activation of a shared neural network involved in movement regulation but not directly linked to interhemispheric interaction mechanisms (Horenstein et al., 2009; Vasilyev et al., 2016). The other hypothesis suggests that bilateral activation might be related to motor synergies formed as a result of individual experience, leading to stereotypical bilateral coactivation in certain movements, such as finger movements. In the human motor experience, bilateral finger movements may have been prevalent due to the need for precise coordination of bimanual finger motor skills (Vasilyev et al., 2016). The ipsilateral ERD component observed in bilateral EEG patterns may reflect the excitability of uncrossed pathways projecting to the muscles, which was supported by TMS-induced MEP assessment (Hasegawa et al., 2017).

EEG responses can also vary depending on the degree and location of lesions, such as those occurring after cerebral stroke, as well as the duration from the stroke to the start of rehabilitation (Stepień et al., 2011; Kaiser et al., 2012; Park et al., 2016). In healthy participants, contralateral ERD observed during motor execution or imagery may be decreased (Stepień et al., 2011) and even shift to the ipsilateral hemisphere (Kaiser et al., 2012; Park et al., 2016). Moreover, the strength of ERD in the contralesional hemisphere depends on the degree of stroke-induced impairment (Kaiser et al., 2012). Our cases demonstrate that the expression of ERD patterns and BCI-classification accuracy were more favorable for patients with a shorter period from the stroke to the start of rehabilitation (Table 1).

In our study, during rehabilitation BCI-sessions in Case 31, we observed ipsilateral ERD (e.g., contralesional) in both μ- and β-frequency bands, which is consistent with findings from previous studies (Kaiser et al., 2012; Park et al., 2016). This activation pattern reflects the transfer of sensorimotor functions to an unaffected hemisphere.

In case 32, there was a prominent ERD observed in both μ- and β-frequency ranges, localized in the contralateral hemisphere to the paralyzed hand. Importantly, the impairment did not affect sensorimotor cortical areas but was instead localized in the vertebro-basilar system. This may explain the prominent contralateral ERD observed and the successful BCI-control achieved during the rehabilitation sessions.

Peculiar ERD activation patterns were observed in Case 42. During MI-based BCI rehabilitation of the left hand, bilateral activation was observed in the μ-range, while ipsilateral lateration (e.g., contralesional) was evident in the β-range. It should be noted that for the μ-rhythm, ERD was predominantly pronounced in the contralateral (e.g., ipsilesional hemisphere). Based on the source localization of the sensorimotor components, μ-rhythm localization is associated with the somatosensory cortex, whereas β-rhythms are linked to the motor cortex (Salmelin and Hari, 1994; Frolov et al., 2014). Park et al. (2016) reported ipsilateral ERD in the β-range for patients with M1 impairments, which supports these observations. Consequently, it is possible to suggest that in Case 42, there were varying degrees of impairment in the somatosensory and motor cortices, leading to distinct ERD lateralization patterns. Following this line of reasoning, one may assume that the left somatosensory cortex was less impaired compared to the primary motor cortex, resulting in a bilateral pattern with contralateral dominance (Figure 4).

### 5.3 Limitations

This study has several important limitations. First, the sample size was small and unbalanced, and the work was designed as a retrospective exploratory case series. Accordingly, the findings should be interpreted as feasibility-oriented observations rather than evidence of clinical efficacy.

Second, the cohort was heterogeneous with respect to lesion location, time since stroke, and clinical status, which may have contributed to the observed inter-individual variability in ERP and ERD patterns. Neurophysiological results were therefore interpreted at the individual level without group-level inference.

Third, only descriptive statistical analyses were performed in accordance with CARE guidance. Metrics such as Cohen's kappa were used solely to demonstrate BCI control above chance and should not be interpreted as indicators of therapeutic effect.

Fourth, all participants continued standard-of-care rehabilitation during the study period; therefore, any functional changes cannot be attributed exclusively to the BCI intervention.

Furthermore, due to the retrospective nature of this analysis, formal patient-reported perspectives on the intervention were not systematically collected.

Finally, interpretation of ipsilateral ERD patterns should be approached cautiously, as such activity may reflect compensatory or non-specific cortical processes rather than adaptive motor recovery.

Future prospective studies with larger and more homogeneous cohorts, standardized follow-up, and controlled designs are required to determine the clinical relevance of the proposed approach.

## 6 Conclusion and future perspective

In recent years, progress has been made in using BCIs to empower people with disabilities, enabling them to control prosthetic devices (Masiero et al., 2014) and interact with virtual reality avatars (Laver et al., 2017). By synergistically merging traditional rehabilitation modalities with intelligent therapies, this approach represents a feasible strategy for exploring new paradigms in motor dysfunction rehabilitation. Our study presented several results from the rehabilitation of five patients who underwent rehabilitation with a BCI-driven robotic orthosis providing passive movements of the paretic limb and a VR environment containing visual goals to achieve. Such a gamified rehabilitation paradigm evokes visuomotor transformation cortical mechanisms, facilitating the potential for holistic recovery.

Traditional wearable exoskeletons, a staple in upper limb rehabilitation, are often limited to fine movements of the upper limb and rarely consider extensive movements such as those involving the shoulder or femur joints, which at the same time play a critical role in daily activities (Molteni et al., 2017). Our system uses a robotic assistant to provide abduction or adduction of the shoulder joint. Such extensive movements are commonly rehabilitated during the initial post-stroke phases, so the approach presented here aptly addresses the requirements of early rehabilitation (however, there are no limitations to the application of the paradigm discussed here, which induces the visuomotor transformation process for fine motor skills rehabilitation).

In particular, the findings from subject feedback converge toward a consensus: the symbiotic integration of BCI, robotic orthosis, and virtual environment may support motor rehabilitation processes and enhance patient engagement. This is in line with a growing body of evidence suggesting the multifaceted benefits of BCI systems that extend beyond motor rehabilitation into domains of cognitive function and emotional state of stroke patients. Changes in these areas may synergistically promote restorative neuroplasticity in motor cortical areas (Mane et al., 2020; Hougaard et al., 2021).

## Data Availability

The datasets presented in this article are not readily available because of ethical and privacy restrictions. Requests to access the datasets should be directed to the corresponding author.
