# Supplementary material for: Case Report: post-stroke rehabilitation with a visuomotor transformation-based brain-computer interface
Source: Front Hum Neurosci. 2026 Apr 14;20:1774409. doi: 10.3389/fnhum.2026.1774409 (PMC13121322; doi:10.3389/fnhum.2026.1774409)
Supplement: Supplementary file 1 [file Table_1.docx]

**Supplementary Table S1.**

Detailed muscle strength assessment using the Medical Research Council (MRC) scale across upper limb muscle groups. Values are reported for baseline and post-intervention assessments, along with absolute changes (Δ). All patients demonstrated changes across multiple muscle groups, with more pronounced gains observed in distal muscles (wrist and finger flexors/extensors).

| **Muscle Group** | **Timepoint** | **Case 23** | **Case 26** | **Case 31** | **Case 32** | **Case 42** |
| --- | --- | --- | --- | --- | --- | --- |
| Shoulder flexors | Baseline | 2 | 2 | 0 | 2 | 2 |
|  | Post-intervention | 3 | 3 | 1 | 2 | 3 |
|  | Δ | +1 | +1 | +1 | 0 | +1 |
| Shoulder extensors | Baseline | 2 | 2 | 0 | 2 | 2 |
|  | Post-intervention | 3 | 3 | 1 | 2 | 3 |
|  | Δ | +1 | +1 | +1 | 0 | +1 |
| Forearm flexors | Baseline | 1 | 3 | 0 | 1 | 2 |
|  | Post-intervention | 2 | 3 | 1 | 2 | 3 |
|  | Δ | +1 | 0 | +1 | +1 | +1 |
| Forearm extensors | Baseline | 1 | 3 | 0 | 1 | 2 |
|  | Post-intervention | 2 | 3 | 1 | 2 | 3 |
|  | Δ | +1 | 0 | +1 | +1 | +1 |
| Forearm pronation | Baseline | 1 | 3 | 0 | 1 | 2 |
|  | Post-intervention | 2 | 3 | 1 | 2 | 3 |
|  | Δ | +1 | 0 | +1 | +1 | +1 |
| Forearm supination | Baseline | 1 | 3 | 0 | 1 | 2 |
|  | Post-intervention | 2 | 3 | 1 | 2 | 3 |
|  | Δ | +1 | 0 | +1 | +1 | +1 |
| Wrist flexors | Baseline | 0 | 2 | 0 | 1 | 1 |
|  | Post-intervention | 2 | 2 | 1 | 2 | 2 |
|  | Δ | +2 | 0 | +1 | +1 | +1 |
| Wrist extensors | Baseline | 0 | 2 | 0 | 1 | 1 |
|  | Post-intervention | 2 | 2 | 1 | 2 | 2 |
|  | Δ | +2 | 0 | +1 | +1 | +1 |
| Finger flexors | Baseline | 0 | 2 | 0 | 1 | 1 |
|  | Post-intervention | 2 | 2 | 1 | 2 | 2 |
|  | Δ | +2 | 0 | +1 | +1 | +1 |
| Finger extensors | Baseline | 0 | 2 | 0 | 1 | 1 |
|  | Post-intervention | 2 | 2 | 1 | 2 | 2 |
|  | Δ | +2 | 0 | +1 | +1 | +1 |
